# Interaction Networks of Prion, Prionogenic and Prion-Like Proteins in Budding Yeast, and Their Role in Gene Regulation

**DOI:** 10.1371/journal.pone.0100615

**Published:** 2014-06-27

**Authors:** Djamel Harbi, Paul M. Harrison

**Affiliations:** Department of Biology, McGill University, Montreal, Quebec, Canada; University of South Florida College of Medicine, United States of America

## Abstract

Prions are transmissible, propagating alternative states of proteins. Prions in budding yeast propagate heritable phenotypes and can function in large-scale gene regulation, or in some cases occur as diseases of yeast. Other ‘prionogenic’ proteins are likely prions that have been determined experimentally to form amyloid *in vivo*, and to have prion-like domains that are able to propagate heritable states. Furthermore, there are over 300 additional ‘prion-like’ yeast proteins that have similar amino-acid composition to prions (primarily a bias for asparagines and glutamines). Here, we examine the protein functional and interaction networks that involve prion, prionogenic and prion-like proteins. Set against a marked overall preference for N/Q-rich prion-like proteins not to interact with each other, we observe a significant tendency of prion/prionogenic proteins to interact with other, N/Q-rich prion-like proteins. This tendency is mostly due to a small number of networks involving the proteins NUP100p, LSM4p and PUB1p. In general, different data analyses of functional and interaction networks converge to indicate a strong linkage of prionogenic and prion-like proteins, to stress-granule assembly and related biological processes. These results further elucidate how prions may impact gene regulation, and reveal a broader horizon for the functional relevance of N/Q-rich prion-like domains.

## Introduction

Yeast prions are propagating altered states of proteins that can be transmitted sustainably into yeasts cell during budding, mating or laboratory infection protocols. The first well-characterized yeast prions, that underlie the [PSI+] and [URE3] prion states, are propagating amyloid forms of the proteins Sup35p and Ure2p. [PSI+] arises from the propagation of an amyloid form of Sup35p, part of the translation termination complex. Thus, formation of [PSI+] prions reduces the efficiency of translation termination and increases levels of nonsense-codon readthrough [1], [2]. Such readthrough has been demonstrated to be a potential mechanism to uncover cryptic genetic variation [3], [4]. [URE3], the prion form of the nitrogen catabolism protein Ure2p, functions to upregulate poor nitrogen source usage, even when rich sources are available [5]–[7]. Some prion variants may also be considered as diseases of budding yeast [8], [9].

A common compositional feature of almost all of the well-characterized yeast prions is a tract with a bias for asparagine (N) or glutamine (Q) residues. Bioinformatic surveys of the yeast proteome have demonstrated the existence of hundreds of proteins with ‘prion-like’ domains, *i.e*., domains with a pronounced bias for N and/or Q [10]–[12], with similar numbers of prion-like proteins observed in a wide variety of fungi [11]. Evolutionary analysis showed that the [PSI+] prion domain is conserved across fungal clades that diverged more than 1 billion years ago, with only eight other budding yeast proteins showing similar, phylogenetically deep patterns of bias conservation; the [URE3] prion domain is unique to *Hemiascomycota*, with different parts of it demonstrating purifying selection and frequent N to Q bias switching between clades [11], [13].

The compositional pecularities of known prion domains were used to train a hidden Markov model to derive a protein target list for testing for prion-forming ability [14]. Tests for *in vitro* and *in vivo* amyloid formation were combined with a Sup35 prion assay, wherein predicted prion-forming domains were fused to the C-terminal part of the Sup35p protein (the protein that underlies the [PSI+] prion), and these constructs tested for the ability to produce [PSI+]-like states in yeast cells [14]. About twenty previously uncharacterized ‘prionogenic’ proteins were identified. The positive and negative results (*i.e*., proteins that failed to make amyloid and a [PSI+]-like state in the prion assay) from this survey have been recently used to train an algorithm, PrionScan, that predicts prion domains bioinformatically [15]. ‘Scrambled’ forms of the Ure2p and Sup35p prion-determinant domains that maintain the amino acid composition, can form prions in budding yeast, indicating that prion formation is primarily dependent on amino-acid composition, and not specific sequence features [16], [17]. Building on these analyses, an amino-acid propensity scale for prion formation was developed, and incorporated into the PAPA method for prion prediction [18], [19]. Fiumara and co-workers [20] showed that a subset of prions and disease amyloids contain predicted coiled-coil regions, and need to form coiled-coil protein interaction domains to aggregate efficiently. An examination of transcription factor networks containing yeast prions showed that the regulons of three well-characterized prion-forming transcription factors Cyc8, Mot3, and Sfp1 overlapped at only two genes, one of which was FLO11, a major determinant of multicellularity and cell adhesion [21]. They further demonstrated experimentally that the [MOT3+] prion governs the acquisition of multicellularity in budding yeast [21].

Here, we perform a bioinformatic analysis of sets of prion, prionogenic and prion-like proteins of *Saccharomyces cerevisiae*, focusing on their functional and interaction networks. We observe strikingly significant enrichments of prion-like proteins, in the interactor lists of prion/prionogenic proteins. After dissecting this data in detail, we find two specific gene-regulatory networks of proteins that are significantly enriched in prion, prionogenic and prion-like proteins, and discuss their implications for prion biology. Overall, several data analyses converge to indicate a complex networked role for prion/prionogenic and prion-like proteins in stress granule assembly and related processes.

## Methods

### Data sets of prion, prionogenic and prion-like sequences

Data sets of prion, prionogenic and prion-like sequences were derived from the PrionHome database [22], [23]. This database contains data lists culled from the analysis in Harrison, *et al*. [11], [12] and Alberti, *et al*. [14]. We analyzed four sample sets of proteins, as follows:

- Known Prions (denoted KPs);
- Experimental Prionogenic Domains (EPDs);
- Experimental Prion Negatives (EPNs);
- Bioinformatic N/Q-rich Prion-like Proteins (NQPs).

**Figure 1:**
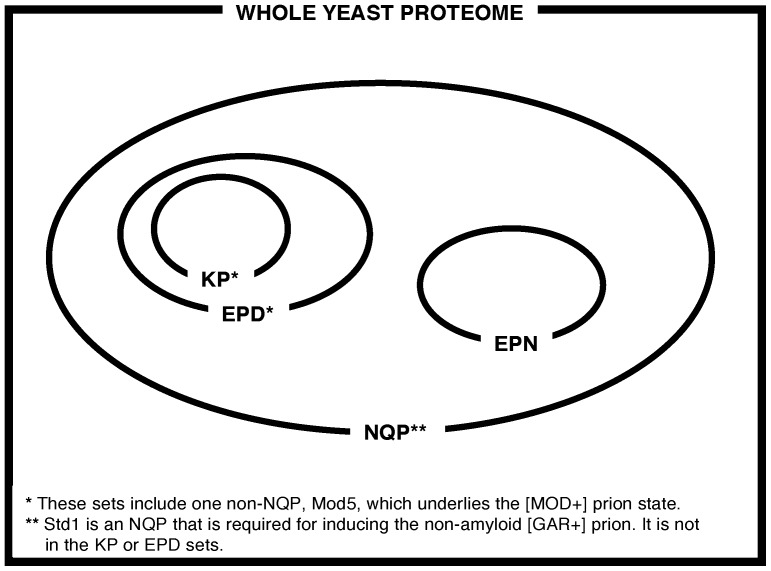
Schematic representation of the relationship between the four data sets KPs, EPDs, EPNs and NQPs.

A schematic representation of the relationships between these four data sets is shown (Figure 1).

Specifically, the *Known Prions* (KP) list comprises proteins that have been relatively well-characterized as amyloid-based prions. These are (UniProt IDs and standard names): P05453, Sup35p; P07884, Mod5p; P09547, Swi1p; P14922, Cyc8p; P23202, Ure2p; P25367, Rnq1p; P32432, Sfp1p; Q08972, New1p; P54785, Mot3p; Q02629, NUP100p).

Only one of these KP proteins is non-N/Q-rich (Mod5p). Also, one of the proteins that we include as a KP could be placed in the EPD set (New1p), since only constructs containing a large prion-determinant New1p subsequence have been shown to propagate prions; however, we included it in the KP set since, like some other KP cases, it has been shown to propagate through the cytoduction technique [23]. Exclusion of these two proteins from the KP data set only marginally affects the enrichment results reported, since they do not interact with any NQPs. Also, both of the proteins have the lowest intrinsic protein disorder content of the KP set (intrinsic disorder sequence fractions, for Mod5p  = 0.14 and for New1p  = 0.13), so the analysis of KPs for protein disorder would not affected significantly by their removal from the KP set either.

The set of *Experimental Prionogenic Domains* (denoted EPDs) comprises the Known Prions *plus* a set of yeast sequences from the analysis of Alberti, *et al*. [14] that have been shown to be likely prions, through a SUP35C prion assay (in conjunction with evidence for *in vivo* amyloid formation by the full-length proteins from the other assays). In the SUP35C prion assay, predicted prion-forming domains are fused to the C-terminal part of the Sup35p protein (the protein that underlies the [PSI+] prion), and these constructs are tested for the ability to produce [PSI+]-like states in yeast cells. The candidate prion-forming domains were predicted to be prionogenic using a Hidden Markov Model algorithm trained on known prion domain cases [14]. The sequences added to the KPs to make the EPD list are: P14907, Nsp1p; P18494, Gln3p; P32588, Pub1p; P32770, Nrp1p; P38180, YBL081W; P38216, YBR016W; P38429, Sap30p; P38691, Ksp1p; P40070, Lsm4p; P40356, Pgd1p; P53894, Cbk1p; Q05166, Asm4p; Q08925, Mrn1p; Q12139, YPR022C; Q12221, Puf2p; Q12224, Rlm1p; Q12361, Gpr1p.

From the same experiments, we made a list of *Experimental Prion Negatives* (EPNs), which are proteins that failed to provide positive results for all of the assays for prion activity in the analysis of Alberti, *et al* [14]. These are: P11746, Mcm1p; P14680, Yak1p; P22082, Snf2p; P23291, Yck1p; P25339, Puf4p; P32505, Nab2p; P32896, Pdc2p; P32900, Skg6p; P38080, Akl1p; P39081, Pcf11p; P43572, Epl1p; P45978, Scd6p; P53617, Nrd1p; P53829, Caf40p; Q03761, Taf12p; Q05785, Ent2p; Q06251, YLR177W; Q12124, Med2p.

The data set of Bioinformatic N/Q-rich Prion-like Proteins (NQPs), are a set of 354 proteins that have N/Q-rich prion-like domains in them, either predicted using the compositional bias criteria in Harrison, *et al*. [11], [12], [23] or the Hidden Markov Model (HMM) used in Alberti *et al*. [14], which was trained on the first four known prion determinant domains (prion-like domains predicted by this algorithm that do not have an N/Q bias were not included in the set) (Text S1). As described in Harrison, *et al*. [11], N/Q-rich prion-like domains were determined using the LPS compositional-bias binomial probability minimization algorithm (with a maximum binomial P-value threshold of 1×10^-10^). Binomial probability thresholds were derived from analyzing KP determinant domains. These prion-like domains are biased purely for N and/or Q residues, or for N and/or Q residues with a subsidiary compositional bias for Y, S or G (with P-value <1×10^−4^), and do not have contributing biases from charged residues (D, E, R or K) or major hydrophobic residues (V, I, L or M), with P-value <1×10^−4^.

The proteins predicted to be candidate prions by the Alberti et al. HMM [14] contain some that do not have a predominant N/Q, N or Q compositional bias. The compositional criteria for such non-N/Q prions in budding yeast have not been yet elucidated; such compositional criteria have however been thoroughly investigated experimentally for the N/Q-rich type of prion over the past decade or more. We thus chose to focus on the NQP proteins for these enrichment analyses.

The NQPs are a list of proteins into which we can probe deeper to analyze bioinformatically the functional characteristics of such proteins and their protein interactions. We have noted that five of the ten relatively well-characterized prions of the KP set do not (P54785, Mot3; P07884, Mod5; P14922, Cyc8; P32432, Sfp1; Q02629, NUP100) did not produce positive results for the Alberti, *et al*. SUP35C prion assay, when tested [14]. This suggests that there may be a further cohort of untested prionogenic proteins in the NQP list.

### Gene Ontology analysis

We examined the Gene Ontology (GO) [24] ‘biological process’ terms of the sets of proteins (Text S2). To assess enrichment of specific GO terms in the sets of proteins, we used hypergeometric probability (with initial P-value threshold of 0.05, corrected for multiple hypothesis testing using the Holm-Bonferroni method over all sets examined for 2487 biological process GO terms) to assess significance of enrichments:
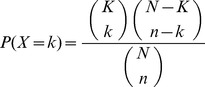
We summed over all values of P(X≥k). *K* is the total number of occurrences of a GO term in the background population (the whole yeast proteome), *N* is total number of yeast proteins, *k* is the number of occurrences of a GO term in a sample data set (KP, EPD, EPN and NQP sets), and *n* is the size of this data set.

So that we can home in on GO biological process categories that have high *membership* from the EPD, EPN and NQP sets, we ranked significantly enriched categories, according to how many yeast proteins annotated with the GO category appear in the protein sets examined. These enrichments were then collapsed to the most specific GO category, if multiple enrichments were due to the same list of proteins, and if the most specific GO category yielded a lower P-value (Text S3). We looked for GO categories most of whose annotated proteins in yeast appear in the EPD, EPN and NQP sets (*i.e*., greater than half of the yeast proteins annotated for the GO category). This procedure helps us to find GO biological process categories which have a relatively small number of annotations in budding yeast, but which may be especially relevant to prion biology, or to the function of N/Q-rich domains.

In addition, we examined for significant enrichments of GO biological process terms in the protein interactions of the KP, EPD, EPN and NQP sample data sets (Text S4). The interacting proteins are not considered individually, but only as GO terms (*i.e*., if protein A interacts with both protein B and protein C, and B and C are both assigned to GO term X, then we consider it as one interaction between protein A and GO term X). For this calculation, the terms in the hypergeometric probability equation are:

*N* =  number of interactions between any protein and GO term for the whole yeast proteome;

*K* =  number of interactions between any protein and a specific GO term for the whole yeast proteome;

*n* =  total number of interactions between any protein and GO term for the sample data set;

*k* =  number of interactions between any protein and a specific GO term for the sample data set.

As above, we applied a Holm-Bonferroni correction for multiple hypotheses.

### Sequence annotation

We used the DISOPRED2 program [25] with default parameters, to annotate intrinsically disordered regions. PAIRCOIL2 (default parameters) [26] and COILS (with less strict parameter values of P>0.5 and window size  = 21) [27] were applied to predict coiled-coil regions. Lists of proteins that contain protein-binding domains (associated with the GO term for ‘protein-binding’, here termed PBDs) were obtained from InterPro [28]. The program LPS was used to annotate compositionally-biased regions in the yeast proteome [29], [30]. Sequence regions that have amino-acid biases with binomial P-value ≤1×10^−10^ were considered in this analysis, since known yeast prion proteins (with the exception of the non-N/Q-rich Mod5) contain regions of bias for glutamine and asparagine of at most this P-value.

### Protein interaction analysis

Binary protein interaction data was downloaded from the IntAct database [31]. Protein interaction lists for the various sets of prion, prionogenic and prion-like proteins were collated. To assess for enrichment/depletion in these protein interaction lists, we counted up the number of interactions between the first ‘sample’ set (from which the interaction list is derived) and the second ‘enrichment/depletion’ set (which is used to test for enrichment or depletion). For the hypergeometric probability formula given above:

*N* =  total number of protein interactions for the yeast proteome;

*K* =  total number of interactions involving an enrichment/depletion set protein;

*n* =  total number of interactions for the sample set of yeast proteins;

*k* =  total number of interactions for the sample set of yeast proteins, involving an enrichment/depletion set protein.

We performed statistical tests using hypergeometric probability and an initial P-value threshold of 0.05, corrected for multiple hypothesis testing using the Holm-Bonferroni method, over all the tests performed (totalling 72 tests).

## Results/Discussion

We have defined sets of known prions (KP), experimental prionogenic proteins proteins that are either KPs or that contain a domain that has been shown experimentally to make a likely prion (EPDs), experimental prion negatives (EPNs) that have been shown experimentally to be unlikely to form prions, and bioinformatically derived N/Q-rich prion-like proteins (NQPs) (Figure 1). All of these four data sets contain regions that are rich in asparagine and/or glutamine residues.

Our main objective is to thoroughly analyze the protein interaction networks of these sets of prion, prionogenic and prion-like proteins. Do these proteins tend to interact preferentially with each other, or with other groups of proteins? If this is the case, can we extract protein networks and protein network features that may be important to prion biology? Before that, however, we overview the functional associations of the data sets, and briefly examine the protein disorder content of these protein sets, since this is information that is important for interpreting the protein interaction data.

### Overview of biological processes of prion, prionogenic and prion-like proteins and their protein interactions

To assess which biological processes the KP, EPD, EPN and NQP data sets function in, we examined for enrichments of Gene Ontology biological process terms. The EPDs and EPNs do not have any biological process enrichments that are significant after multiple hypothesis correction (detailed in Text S2 and *Methods*). The KPs do not have any enrichments with P-value <0.05. The most significant of the EPD and EPN enrichments indicate preferences for EPDs to function in (positive) regulation of transcription (in response to stress), and in response to abiotic stimuli. These enrichments are not observed for EPNs.

NQPs are broadly functional in (positive) transcriptional regulation, with specific enrichments in response to stress (Text S2). Most interestingly, we find many GO *biological process* categories with *high membership* of NQPs (calculated as described in *Methods*) that are linked to stress granule assembly and to response to stress, specifically osmotic stress and salt stress (Text S3). (The proteins causing these enrichments are not members of the KP, EPD or EPN data sets.) Stress granules are aggregations of protein and RNA that form inside cells when they are in stressful environments. A linkage of NQPs to GO function categories related to ribonucleoprotein assemblies has been noted previously, amongst many other linkages [32]. The links of EPDs and NQPs to environmental stress response are interesting in light of the hypothesized general role of prions in enabling large-scale regulatory shifts that may aid in responding to environmental change [2]. There is direct experimental evidence for such an environmental stress response role for the [PSI+], [MOD+] and [MOT3] prions [3], [4], [14], [21], [33].

In addition, we examined for biological process enrichments in the *interaction*s of the prion, prionogenic and N/Q-rich prion-like proteins (NQPs) (Text S4). The NQPs interact highly significantly with stress granule assembly proteins (P = 6×10^−11^). The general processes of ‘regulation of mRNA stability’ and ‘negative regulation of translation’ (which are associated with stress granule assembly) also provide significant interaction enrichments.

### Prionogenic proteins have very high levels of intrinsic disorder

**Figure 2:**
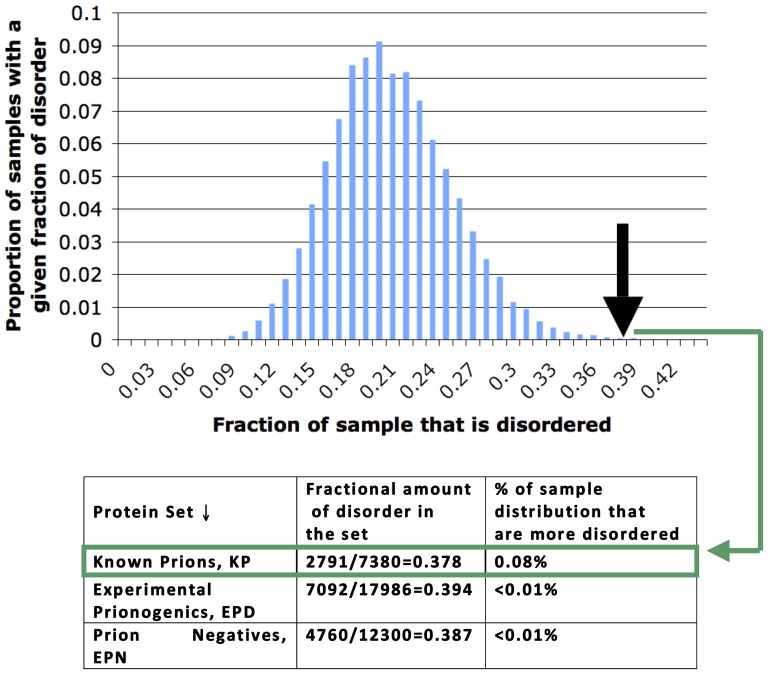
Disorder content of known prions (KPs), experimental prionogenic domains (EPDs) and prion negatives (EPNs). Monte Carlo samples (n = 10000) of the same total protein length as each of the three data sets were made and the total amount of disorder (from the DISOPRED2 program [25]) was annotated. A fractional piece of one protein was used to make up the exact residue count for the sample size. The plot shows the distribution of disorder content for these samples for the KP set. The actual observed value is indicated by an arrow. The % of samples that have greater disorder than the observed value for each data set is indicated in the table below the histogram.

Intrinsic disorder occurs where a protein or protein domain remains natively unfolded, for at least part of its functional biological role. Intrinsic disorder is an important factor in the interaction behaviour of a protein. Certain types of intrinsically disordered proteins tend to interact with each other, and in some biological processes they are important interaction hubs [34], [35]. Here, we have analyzed the overall intrinsic disorder content of the whole protein sequences of the KP, EPD and EPN data sets (not just there known or predicted prion determinant sequences), using the DISOPRED2 program [25]. We observe that these data sets have exceptionally high overall intrinsic disorder content relative to the whole yeast proteome (Figure 2). Very high intrinsic disorder content is thus a defining characteristic of the known budding-yeast N/Q-rich prion/prionogenic protein data sets. The fact that EPNs also have such high disorder content indicates that this is likely a major property of yeast sequences that was extracted by the Hidden Markov Model algorithm trained on the first four known yeast prion domains, that was used to identify prion candidates to experiment on [14].

### Highly significant preferences in protein interaction networks of prion, prionogenic and prion-like proteins

**Figure 3:**
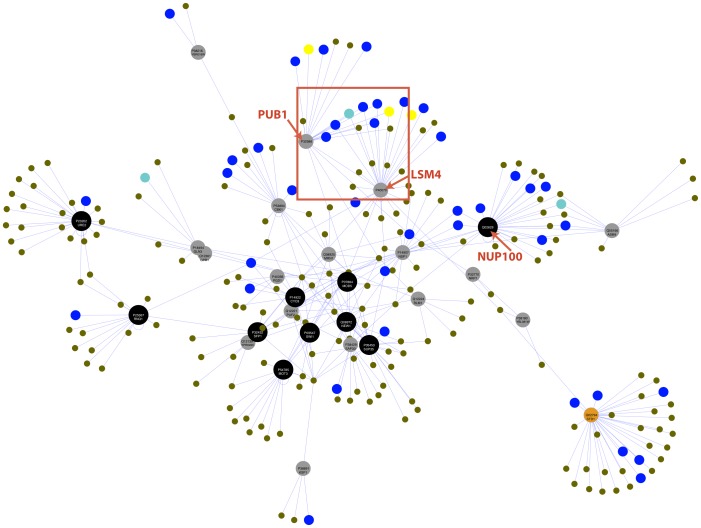
The protein interaction network for the EPD data set. We drew this original picture using the publicly distributed Cytoscape tool that can be used for depicting networks [49], with the data sets of protein interactions that we derived (as described in *Methods*) as input. We have coloured the nodes as follows: --- known prions (KP)  =  **BLACK**; --- other proteins in the EPD data set  =  **GREY**; --- EPN data set  =  **YELLOW**; --- NQPs that are also prion predictions made using the PrionScan algorithm  =  **CYAN**; --- any other NQP  =  **DARK BLUE**; --- other interactors  =  **BROWN**. The non-amyloid prion accessory protein STD1 that underlies the [GAR+] prion [50] (Q02794) is part of the NQP data set that we derived, since it has an N/Q-rich domain. We have coloured its node at the lower right of the network, **ORANGE**. The prion/prionogenic proteins are labelled with their UniProt accessions and standard gene names. The three EPD hubs are pointed out with red arrows. A red box surrounds common interactors between the LSM4 and PUB1 proteins.

We investigated whether there is any significant evidence for preferential interaction of prion (KP), prionogenic (EPD), prion negative (EPN) and N/Q-rich prion-like (NQP) proteins in their lists of protein interactors. The networks of protein interactors for the KP and EPD data sets comprise many linkages between KP, EPD and NQP data set members (as evidenced for the EPD interaction network in Figure 3). To quantify these linkages, we looked for enrichments and depletions of various data sets in the interactor lists of the prion, prionogenic and N/Q-rich prion-like protein sets under study, using hypergeometric probability (Table 1; see *Methods* for details).

**Table 1:** Enrichments for different sets of sequences in the interactor lists for prion, prionogenic and prion-like proteins in budding yeast*.

*Are these sets enriched in the lists of interactors of the sets listed below? →*	KP (10/5796 proteins) *(152/36467 interactions)*	EPD (27/5796 proteins) *(314/36467 interactions)*	NQP (354/5796 proteins) *(4405/36467 interactions)*	EPN (18/5796 proteins) *(259/36467 interactions)*	Disordered proteins (>0.5 disordered) not in NQP data set (251/5796 proteins) *(2432/36467 interactions)*
**KP**	1/152 (NS)	5/152 (0.010)†	20/152 (NS)	1/152 (NS)	6/152 (NS)
**EPD**	5/314 (0.010)†	7/314 (0.019)†	**63/314 (0.000034)**	7/314 (0.0074)†	9/314 (*0.00083, depletion*)
**EPN**	1/259 (NS)	7/259 (0.0074)†	46/259 (0.0050)†	3/259 (NS)	7/259 (0.0013, depletion)†
**NQP**	20/4405 (NS)	**63/4405 (0.000034)**	251/4405 *(<1e−30, depletion)*	46/4405 (0.0047)†	*162/4405 (1e−20, depletion)*

*The interactor lists are in the rows of the table, and the sets that are tested as enriched/depleted or not, are in the columns. These sets are explained in the main text. At the head of each column is given the total number of proteins of each set type, and the total number of interactions involving them. In each cell, is given the number of interactors that are members of the sets tested as enriching/depleted, expressed as a fraction of the total number of interactors. In brackets is given the hypergeometric probability for this enrichment/depletion, with NS for non-significant (P-value threshold  = 0.05). Values that are significant enrichments after Holm-Bonferroni correction are in bold, significant depletions in italics.

† These P-values become non-significant after a Holm-Bonferroni correction over all tests performed (totalling 72).

With the whole yeast interactome as a background population, we observed one significant enrichment, for EPD←→NQP interactions (Table 1). This is striking when compared to a significant depletion in NQP←→NQP interactions overall. Other mild enrichments are observed for KP←→EPD, EPD←→EPD, EPN←→EPD, EPN←→NPQ and EPN←→EPN interactions. However, these are not significant after multiple-hypothesis correction.

To home in on the most important interaction networks of EPDs and NQPs that underlie this EPD←→NQP interaction preference, we looked at the EPDs that interact the most with NQPs. Three EPDs clearly stand out, interacting with >10 NQPs (the other EPDs interact with on average ∼1.4 NQPs, with the next largest number of NQP interactors being 4). These are:

- U6 snRNA-associated Sm-like protein LSM4 (P40070), with 14/31 interactors being NQPs (P-value = 4×10^−6^);
- nuclear and cytoplasmic polyadenylated-RNA-binding protein PUB1 (P32588), with 13/20 interactors being NQPs (P-value = 4×10^−8^);
- nucleoporin NUP100 (Q02629) with 10/28 interactors being NQPs (P-value = 8.5×10^−4^).

The P-values are significant after correction. The networks of these three hubs are discussed in more detail below. Given that a quarter of the EPD interactions (78/314) are for these hubs, we separated the EPD data into hub and non-hub interactions to check whether the EPD←→NQP enrichment is due mostly to these hubs. This seems to be the case (Table 2).

**Table 2:** Enrichments of proteins with and without protein-binding domains (PBDs), in the interactor lists of the EPDs*.

*Are these sets enriched in the interactors of the sets listed below? →*	NQPs *(4405/36467 interactions)*	Proteins containing PBDs but not NQP domains** *(19481/36467 interactions)*	Proteins containing NQP domains but not PBDs *(2877/36467 interactions)*	Proteins containing NQP domains and PBDs *(1631/36467 interactions)*	Disordered proteins (>0.5 disordered) not in NQP data set *(2432/36467 interactions)*
**EPD**	**63/314 (0.000034)**	89/314 *(9e−20,depletion)*	**48/314 (0.0000079)**	15/314 (NS)	*9/314 (0.00083, depletion)*
**EPD hubs**	**36/78 (8e−14)**	8/78 *(9e−16,depletion)*	**27/78 (2e−11)**	9/78 (0.006)†	0/78 (0.005, depletion)†
**EPD non-hubs**	27/236 (NS)	81/236 *(1e−09,depletion)*	21/236 (NS)	6/236 (0.048)†	9/236 (0.022, depletion)†

*As for Table 1.

**Proteins containing PBDs are those containing predicted coiled-coil regions or protein-binding domains defined specifically as such, in InterPro (see *Methods* for details). The enrichment of proteins containing PBDs in the list of their own interactors is very highly significant (P = 4e−52).

† As for Table 1.

Because of the significant overall depletion of NQP←→NQP interactions in the yeast interactome, we examined enrichments in the KP, EPD and EPN sets involving the NQP proteins, using the NQP interactome as a background population, instead of the whole yeast proteome (Table S1). With these criteria, after corrections, the EPD←→NQP interaction preference becomes more striking. For the other prion-related data sets, including KPs and the less promiscuous EPD non-hubs, preference for NQP interaction becomes significant, indicating a general preference to interact with other N/Q-rich proteins.

We checked whether the definition of the NQP set had an effect on the observed enrichments of NQPs that we have listed in Tables 1 and S1. To do this, we examined enrichments for the original lists of Alberti, *et al*., prion domain predictions [14], the list of prion-like domains by Harrison, *et al*. [11], [12], and the ‘intersection’ of these two lists (Table S2). The Alberti, *et al*., list contains a small number of non-NQP prion domain predictions. We examined using both the whole proteome, and the NQPs as a background population. The results indicate that the NQP enrichments observed, particularly for EPDs, are robust to the criteria for NQP data set definition. Particularly notable is that the EPD non-hubs have significant enrichments of NQP interactors for the Harrison, *et al*. and ‘intersection’ data sets (Table S2).

Since the KP, EPD and EPN data sets are themselves very highly disordered (Figure 1), we checked whether they interact preferentially with other very highly disordered proteins. To do this, we constructed a data set of 251 highly disordered proteins (that have disordered content comprising >50% of their sequences, annotated using the DISOPRED2 program [25]), and that are not in the NQP set. These proteins are not significantly enriched as interactors of any of the prion, prionogenic or prion-like protein sets, and are in fact significantly depleted in most cases (Tables 1–2). Thus, the NQP interaction enrichments are not due to the most highly-disordered subset of proteins being more interactive with NQPs.

Furthermore, we checked whether co-occurrence in the sequences of various defined ‘protein-binding’ domains (PBDs) may be causing the EPD←→NQP interaction preference (Table 2). These protein-binding domains are derived from the InterPro database, and from predictions of coiled coils in the yeast proteome (see *Methods* for details). We find that there is an obvious preference for the overall EPD set, and also the EPD hubs, to interact with proteins that contain N/Q-rich prion-like domains, but that lack the PBDs. Proteins containing PBDs, but which are not NQPs, are significantly depleted in the EPD interactor lists. Thus, the preferences are unlikely to arise from PBD co-occurrences.

These data indicate that some yeast prion/prionogenic proteins are part of a definable interaction sub-network, involving other proteins that have N/Q-rich prion-like domains. These preferred interactions are not due to either binding to other very highly disordered proteins, or to the presence of protein-binding domains in the sequences. The interactions with NQPs may be mediated, at least in part, by the prion/prion-like N/Q-rich domains. (Of course, such N/Q-rich domain interactions may not be necessary or sufficient for any individual protein interaction.) There are many examples of N/Q-rich domains mediating protein interaction in the literature. For example, Q-rich domains in the transcription factor *Sp1* are involved in interaction with other nuclear proteins and in self-interaction [36]. The protein *Zranb2* in zebrafish, interacts via a Q-rich domain with *Smad1*, a protein important in the regulation of bone morphogenetic proteins [37]. The Q-rich domain of budding yeast *Gal11*, a component of the Mediator complex required for transcriptional activation of many genes, is necessary for interaction with glucocorticoid receptor tau 1 [38]. One should note, however, that the overall significant depletion in NQP←→NQP interactions (Tables 1–2) is evidence against N/Q-rich domains being generic protein interaction modules in general.

### The three KP/EPD hubs

The three major KP/EPD hubs are NUP100p, PUB1p and LSM4p (Figure 3). NUP100 is a prion protein [39]. It is a nucleoporin containing FG (Phe/Gly) repeats. It is part of the nuclear pore complex for transport of mRNAs and proteins across the nuclear membrane. NUP100 functions in many nuclear import/export pathways. Of the ten NQPs that it interacts with, eight are also members of the nuclear pore complex. One of these is also an experimental prionogenic (ASM4), and another (NUP116) is predicted to be prionogenic by the recent PrionScan algorithm [15], which was trained on EPD and EPN data from the analysis of Alberti, *et al*. [14]. In addition to the demonstrated prion-forming ability of NUP100, amyloid-like interactions by individual nucleoporin FG-repeat domains, and by the Nsp1p nucleoporin, have been observed as hydrogel formation [40], [41].

LSM4 is an experimentally-determined prionogenic protein [14]. It is a component of the LSM complexes that function in RNA processing. The set of 14 NQPs that interact with LSM4 are significantly associated with GO biological process categories ‘negative regulation of translation’ (and more specifically ‘negative regulation of translation initiation’), ‘stress granule assembly’ and other related, more general GO categories (Text S5). LSM4 directly interacts with PUB1, the third KP/EPD hub, and with two proteins (PUF4 and SCD6) predicted to be prionogenic by PrionScan [15]. Upon overexpression, LSM4 has been shown to form amyloids that play a key role in elimination of the [PSI+] prion from cells [42]–[44]. Also, ‘P-bodies’, which are cytoplasmic RNA granules that contain translationally repressed ribonucleoproteins, can be formed via the N/Q-rich domain of LSM4 [45].

The nuclear and cytoplasmic polyadenylated RNA-binding protein PUB1 was determined to be prionogenic experimentally [14]. It binds to messenger RNAs during nucleocytoplasmic mRNA transport. The 13 NQPs that interact with PUB1 are significantly linked to GO biological process categories ‘negative regulation of translation’, and ‘regulation of mRNA stability’ (Text S5). Two of the NQPs are PrionScan [15] prionogenic predictions (PUF4 and NAB2, which also binds polyadenylated RNA). Six NQP proteins are common to the networks of LSM4 and PUB1 (Figure 3). LSM4 and PUB1 are thus central in a network of prionogenic and prion-like proteins that functions in stress granule formation and negative regulation of translation.

### A role for prion, prionogenic and prion-like proteins in stress granule assembly and other gene regulation processes

Do any biological processes arise multiple times in the bioinformatic analyses above? We performed enrichment analysis to find biological process GO categories that have high membership of NQPs (Text S3), that are enriched in the protein interactions of NQPs (Text S4), and that are enriched for the interactomes of specific hub proteins that are prion or prionogenic (Text S5). By cross-referencing these three different GO category enrichment sets, we find that ‘stress granule assembly’ is the only GO category that counts significantly amongst all three of them.

Stress granules are aggregations of protein and RNA that form inside cells when they are in stressful environments. Formation of stress granules is likely a protective mechanism for RNAs and their associated proteins. In mammalian cell lines, TIA-1 promotes stress granule assembly through prion-like aggregation in response to stress [46]. This activity is maintained if the N/Q-rich domain of TIA-1 is replaced with the prion determinant domain of budding yeast Sup35p [46]. On the other hand, stress granule component proteins, including PUB1 and PBP1, are specifically recruited to polyglutamine aggregates in a laboratory model sytem [47]. This is hypothesized to contribute to polyglutamine disease pathology [48]. Thus, the detailed role of N/Q-rich domains in stress granule formation remains far from clear.

Generally, the prion/prionogenic protein interactions with NQPs observed in these analyses may be mediated (at least in part) by the N/Q-rich prion/prion-like domains. Although some of the binary interactions detected may be non-functional (*e.g*., random interactions like seed precursors for amyloids, or some other sort of interaction ‘noise’), the specific network structures discovered indicate very non-random network topologies, particularly considering the general significant depletion in NQP←→NQP interactions. Alternatively, rather than being a part of direct protein interaction, the N/Q-rich domains may serve some unknown general function in the GO categories to which the three hubs are linked, *e.g*., a mechanistic role in channel operation (in the case of NUP100), or in the positioning of proteins relative to other protein complexes (in the case of LSM4 and PUB1).

Overall, these results suggest a potential regulatory mechanism of prion formation. By forming prion amyloids, proteins can be taken away from specific functional interactions with other N/Q-rich proteins. Conversely, regulatory protein interactions in networks that include a prion-forming protein might, for example, have a functional role in the sensing of prion propagation efficiency. Some prion variants appear to be beneficial, whereas other prion forms behave as yeast diseases [2], [9]. Thus, such regulatory interactions would have to evolve so that prion formation is avoided in situations where it is detrimental to fitness, but also, enabled in environments where it is beneficial.

### Conclusions

We have shown that there is an interaction preference between prion/prionogenic proteins and other, N/Q-rich prion-like proteins. Although prion/prionogenic proteins are a very highly intrinsically disordered subset of yeast proteins, they tend not to interact with other highly intrinsically disordered proteins that do not contain an N/Q-rich prion-like domain. The interaction preference of prion/prionogenic proteins with N/Q-rich prion-like proteins is due mostly to gene regulatory protein interaction networks involving the LSM4 and PUB1 prionogenic proteins, and the NUP100 prion. In general, N/Q-rich prion-like proteins are significantly unlikely to interact with each other. The specific interaction networks for prion/prionogenic proteins are thus unlikely to comprise non-functional interactions. Several enrichment analyses point towards a general functional linkage of prion, prionogenic and prion-like proteins to stress granule assembly. These results might help in pinpointing further prions, and proteins that are important in prion biology. They have also illuminated the role of N/Q-rich prion-like domains in interaction networks involved at various stages of gene regulation. The strategy of analysis can be applied to other compositionally defined domains to elucidate their roles in biological processes.

## Supporting Information

Table S1 **Enrichments for NQPs in the interactor lists for prion and prionogenic proteins in budding yeast, using the data for NQP interactions as a background.**
(DOC)

Table S2 **Checking the enrichments of various other protein sets containing NQPs.**
(DOC)

Text S1 **Lists of NQP proteins and other data sets analyzed.**
(DOC)

Text S2 **Enrichments in Gene Ontology (GO) biological process categories for the EPD, EPN and NQP data sets.** The format of each line is as follows (the n fields are numbered $1,$2,…,$n and tab delimited): $1 =  data set (KP, EPD, EPN or NQP). $2 =  GO category. $3 =  description of the GO category in words. $4 =  hypergeometric probability of the enrichment. For the NQP set, those GO biological process categories that are significantly enriched after a Holm-Bonferroni correction for multiple hypotheses, are listed. For the other data sets, none of the categories are significantly enriched after the Holm-Bonferroni correction. In these cases, any with P-value < = 1E−4 are listed.
(DOC)

Text S3 **Gene Ontology biological process categories that have high membership, for the EPD and NQP data sets.** The format of each line is as follows (the n fields are numbered $1,$2,…,$n, tab-delimited): $1 =  Number of yeast proteins in the GO category. $2 =  Number of proteins from the set (EPD or NQP) in the GO category. $3 =  the fraction of the yeast proteins in the GO category that are in the set (EPD or NQP). $4 =  GO biological process category. $5 =  description of the GO category in words. $6 =  hypergeometric probability of the enrichment. $7 =  YES or NO for whether the enrichment passes the Holm-Bonferroni correction for multiple hypotheses. All enrichments with raw hypergeometric P-values < = 1E−4 are listed, if also most (> = 0.5) of the yeast proteins in thatGO biological process are included in the data sets. For the NQP set, those that are are significant after a Holm-Bonferroni correction are listed first.
(DOC)

Text S4 **Enrichments in Gene Ontology (GO) biological process categories for the protein interactions of the EPD, EPN and NQP data sets.** The format of each line is as follows (the n fields are numbered $1,$2,…,$n and tab delimited): $1 =  Number of yeast proteins in the GO category. $2 =  Number of proteins in the GO category that are interacted with by the set. $3 =  GO category. $4 =  description of the GO category in words. $5 =  hypergeometric probability of the enrichment. $6 =  YES or NO for whether the enrichment passes the Holm Bonferroni correction for multiple hypotheses. For the NQP set, those GO biological process categories that are significantly enriched after a Holm-Bonferroni correction for multiple hypotheses, are listed. For the other data sets, none of the categories are significantly enriched after the Holm-Bonferroni correction. In these cases, any with P-value < = 1E−4 are listed.
(DOC)

Text S5 **Enriched Gene Ontology (GO) biological process categories for the NQP interactors of the three hubs LSM4, PUB1 and NUP100.** The format of each line is as follows (the n fields are numbered $1,$2,…,$n and tab delimited): $1 =  Number of yeast proteins in the GO category. $2 =  Number of proteins in the GO category that are interactors of the specific KP/EPD protein examined. $3 =  GO category. $4 =  description of the GO category in words. $5 =  hypergeometric probability of the enrichment. The enrichments that are significant after multiple-hypothesis correction with the Holm-Bonferroni method are listed.
(DOC)
