# Supplementary material for: Interaction Networks of Prion, Prionogenic and Prion-Like Proteins in Budding Yeast, and Their Role in Gene Regulation
Source: PLoS One. 2014 Jun 27;9(6):e100615. doi: 10.1371/journal.pone.0100615 (PMC4074094; doi:10.1371/journal.pone.0100615)
Supplement: Table S1 — Enrichments for NQPs in the interactor lists for prion and prionogenic proteins in budding yeast, using the data for NQP interactions as a background. (DOC) [file pone.0100615.s001.doc]

**Table S1: Enrichments for NQPs in the interactor lists for prion and prionogenic proteins in budding yeast, using the data for NQP interactions as a background***

|  | **Proportion of interactions involving each of the listed sets, that are with NQPs**  (251/4405 NQP interactions are NQP-to-NQP) |
| --- | --- |
| **KP**** | **20/152 (0.0002)** |
| **EPD**** | **63/314 (2e-20)** |
| **EPN** | **46/259 (6e-13)** |
| **EPD hubs** | **36/78 (3e-25)** |
| **EPD non-hubs** | **27/236 (0.0002)** |

* The interactor lists are in the rows of the table. At the head of the column is given the total number of proteins of each set type, and the total number of interactions involving them. In each cell, is given the number of interactors that are members of the sets tested as enriching/depleted, expressed as a fraction of the total number of interactors. In brackets is given the hypergeometric probability for this enrichment/depletion, with NS for non-significant (P-value threshold =0.05). Values that are significant enrichments after Holm-Bonferroni correction are in bold, significant depletions in italics.

** Mod5 (P07884) is a KP that is not an NQP; since it does not interact with any NQPs it has no effect on these calculations.
