# Supplementary material for: Interaction Networks of Prion, Prionogenic and Prion-Like Proteins in Budding Yeast, and Their Role in Gene Regulation
Source: PLoS One. 2014 Jun 27;9(6):e100615. doi: 10.1371/journal.pone.0100615 (PMC4074094; doi:10.1371/journal.pone.0100615)
Supplement: Table S2 — Checking the enrichments of various other protein sets containing NQPs. (DOC) [file pone.0100615.s002.doc]

**Table S2: Checking the enrichments of various other protein sets containing NQPs ***

| ***Which of these are enriched in the interaction set ? 🡪*** | **Alberti, *et al.* set** | | **Harrison *et al.* set** | | **Alberti *et al.* and Harrison *et al* intersection set** | |
| --- | --- | --- | --- | --- | --- | --- |
|  | **Using whole proteome as background population**  *(3467/36467interactions)* | **Using NQPs as background**  **population**  *(148/3467)* | **Using whole proteome as background**  **population**  *(4038/36467)* | **Using NQPs as background**  **population**  *(182/4038)* | **Using whole proteome as background**  **population**  *(1745/36467)* | **Using NQPs as background**  **population**  *(43/1745)* |
| **KP interaction set** | 12/152 (NS) | 12/152 (0.027)† | 19/152 (NS) | **19/152 (0.000039)** | 9/152 (NS) | 9/152 (0.0097)† |
| **EPN**  **Interaction set** | 32/259 (NS) | **32/259 (2e-8)** | 32/259 (NS) | **32/259 (8e-8)** | 19/259 (0.044)† | **19/259 (0.0000027)** |
| **EPD interaction set** | 46/314 (0.002)† | **46/314 (6e-15)** | **54/314 (0.00071)** | **54/314 (2e-19)** | **32/314 (0.00005)** | **32/314 (3e-16)** |
| **EPD hubs interaction set** | **30/78 (7e-12)** | **30/78 (3e-22)** | **28/78 (7e-9)** | **28/78 (5e-19)** | **20/78 (5e-10)** | **20/78 (4e-17)** |
| **EPD non-hubs interaction set** | 16/236 (NS) | 16/236 (0.042)† | 26/236 (NS) | **26/236 (0.00001)** | **12/78 (0.00032)** | **12/78 (1e-7)** |

* At the head of the column is given the total number of proteins of each set type, and the total number of interactions involving them. In each cell, is given the number of interactors that are members of the sets tested as enriching/depleted, expressed as a fraction of the total number of interactors. In brackets is given the hypergeometric probability for this enrichment/depletion, with NS for non-significant (P-value threshold =0.05). Values that are significant enrichments after Holm-Bonferroni correction are in bold, significant depletions in italics.

† These P-values are NS after Holm-Bonferroni correction.
