# Supplementary material for: Interaction Networks of Prion, Prionogenic and Prion-Like Proteins in Budding Yeast, and Their Role in Gene Regulation
Source: PLoS One. 2014 Jun 27;9(6):e100615. doi: 10.1371/journal.pone.0100615 (PMC4074094; doi:10.1371/journal.pone.0100615)
Supplement: Text S1 — Lists of NQP proteins and other data sets analyzed. (DOC) [file pone.0100615.s003.doc]

Text S1: Lists of NQP proteins and other data sets

==================================================

analyzed*

========

*Accession IDs from the UniProt database, http://www.uniprot.org

Main NQP data set

-----------------

O13577

P00724

P00724-2

P01119

P02381

P04147

P04386

P04821

P05453

P06245

P06774

P07269

P07272

P08004

P08153

P09547

P09798

P0C2I2

P0C2I9

P10870

P10961

P11746

P11792

P12383

P13186

P13483

P14064

P14680

P14907

P14922

P16649

P17442

P18480

P18494

P18852

P18888

P18899

P19659

P20134

P20486

P20676

P21190

P21192

P21657

P21957

P22035

P22082

P22148

P22149

P22470

P22579

P23202

P23250

P23291

P23292

P23293

P23900

P24276

P24784

P24814

P25042

P25299

P25302

P25339

P25367

P25384

P25502

P25644

P25655

P27514

P27705

P27895

P28003

P29295

P29311

P31380

P31384

P32336

P32350

P32389

P32432

P32478

P32505

P32521

P32588

P32617

P32629

P32644

P32770

P32790

P32831

P32862

P32893

P32896

P32900

P32944

P32945

P33306

P33336

P33338

P33417

P33748

P33749

P34217

P34228

P34730

P34756

P34758

P34761

P35056

P35190

P35210

P35732

P36076

P36102

P36138

P36157

P36168

P36169

P37263

P38042

P38080

P38114

P38129

P38140

P38150

P38153

P38167

P38180

P38201

P38213

P38216

P38236

P38261

P38266

P38277

P38290

P38330

P38429

P38691

P38692

P38699

P38738

P38741

P38781

P38817

P38856

P38873

P38886

P38931

P38956

P38968

P38970

P38996

P39001

P39008

P39016

P39081

P39113

P39517

P39523

P39678

P39719

P39935

P39936

P39937

P39959

P39960

P39962

P39970

P40002

P40038

P40068

P40070

P40095

P40209

P40317

P40343

P40356

P40357

P40358

P40433

P40453

P40455

P40463

P40467

P40473

P40482

P40484

P40485

P40535

P40561

P40578

P40956

P41696

P41813

P41895

P41910

P42845

P43563

P43565

P43572

P43574

P43582

P43592

P43612

P45976

P45978

P46958

P46974

P46997

P47029

P47049

P47069

P47100

P47135

P48016

P48361

P48562

P48568

P48837

P49686

P49687

P50104

P50109

P50875

P50896

P51862

P52593

P52960

P53050

P53104

P53115

P53165

P53185

P53191

P53267

P53281

P53309

P53316

P53438

P53550

P53617

P53829

P53894

P53919

P53939

P53950

P53955

P53968

P54785

Q00245

Q00362

Q00416

Q00539

Q00684

Q00772

Q01477

Q01590

Q01722

Q02100

Q02199

Q02629

Q02630

Q02773

Q02792

Q02805

Q02875

Q02887

Q03088

Q03180

Q03213

Q03306

Q03390

Q03466

Q03482

Q03656

Q03735

Q03761

Q03823

Q03825

Q03833

Q03855

Q03935

Q03957

Q04007

Q04052

Q04195

Q04214

Q04233

Q04545

Q04739

Q04978

Q05166

Q05785

Q05854

Q05934

Q05958

Q06032

Q06251

Q06315

Q06337

Q06449

Q06488

Q06628

Q06639

Q06673

Q06681

Q07084

Q07800

Q07807

Q08045

Q08236

Q08281

Q08471

Q08601

Q08683

Q08732

Q08831

Q08887

Q08925

Q08954

Q08969

Q08972

Q12030

Q12034

Q12057

Q12124

Q12132

Q12139

Q12141

Q12151

Q12171

Q12216

Q12221

Q12224

Q12241

Q12300

Q12361

Q12465

Q12476

Q12489

Q12490

Q12517

Q12518

Q12734

Q12753

Q3E7X8

Q7M4S9

Q99231

Q99257

Q99296

Q99383

Q99395

Harrison, et al. data set

-------------------------

O13577

P00724

P00724-2

P01119

P02381

P04147

P04386

P04821

P05453

P06245

P06774

P07269

P07272

P08004

P08153

P09547

P09798

P0C2I2

P10870

P10961

P11746

P11792

P12383

P13186

P13483

P14064

P14680

P14907

P14922

P16649

P17442

P18480

P18494

P18852

P18888

P19659

P20134

P20486

P21190

P21192

P21657

P21957

P22035

P22082

P22148

P22149

P22470

P23202

P23250

P23291

P23292

P23900

P24276

P24814

P25042

P25299

P25302

P25339

P25367

P25384

P25502

P25644

P25655

P27514

P27705

P27895

P28003

P29311

P31380

P32336

P32350

P32389

P32432

P32478

P32588

P32617

P32629

P32644

P32770

P32790

P32831

P32862

P32893

P32896

P32900

P32944

P32945

P33306

P33336

P33338

P33417

P33748

P33749

P34217

P34228

P34730

P34756

P34758

P34761

P35210

P35732

P36076

P36102

P36138

P36157

P36168

P36169

P37263

P38042

P38080

P38114

P38129

P38140

P38150

P38153

P38167

P38180

P38201

P38213

P38216

P38261

P38277

P38290

P38429

P38691

P38692

P38699

P38738

P38741

P38781

P38817

P38873

P38886

P38931

P38956

P38968

P38970

P38996

P39001

P39008

P39081

P39113

P39523

P39678

P39719

P39935

P39936

P39937

P39959

P39960

P39962

P39970

P40002

P40038

P40068

P40070

P40095

P40209

P40317

P40356

P40357

P40358

P40433

P40453

P40455

P40463

P40467

P40473

P40484

P40485

P40535

P40561

P40578

P41696

P41813

P41895

P41910

P42845

P43563

P43565

P43572

P43574

P43582

P43592

P43612

P46958

P46974

P46997

P47029

P47049

P47069

P47135

P48016

P48361

P48562

P48568

P48837

P49686

P49687

P50104

P50109

P50875

P50896

P51862

P52593

P52960

P53050

P53104

P53115

P53165

P53185

P53191

P53267

P53281

P53316

P53438

P53550

P53829

P53894

P53919

P53939

P53950

P53955

P53968

P54785

Q00245

Q00362

Q00416

Q00539

Q00684

Q00772

Q01590

Q01722

Q02100

Q02199

Q02629

Q02630

Q02773

Q02792

Q02805

Q02875

Q02887

Q03088

Q03180

Q03213

Q03306

Q03390

Q03466

Q03482

Q03656

Q03735

Q03761

Q03823

Q03825

Q03833

Q03855

Q03935

Q03957

Q04007

Q04052

Q04233

Q04545

Q04739

Q04978

Q05166

Q05785

Q05854

Q05934

Q05958

Q06032

Q06251

Q06315

Q06488

Q06628

Q06639

Q06673

Q06681

Q07084

Q07800

Q07807

Q08045

Q08236

Q08281

Q08471

Q08601

Q08683

Q08732

Q08831

Q08887

Q08925

Q08954

Q08969

Q08972

Q12030

Q12034

Q12057

Q12124

Q12132

Q12139

Q12151

Q12171

Q12216

Q12221

Q12224

Q12241

Q12300

Q12361

Q12465

Q12476

Q12489

Q12490

Q12517

Q12518

Q12734

Q12753

Q3E7X8

Q7M4S9

Q99231

Q99257

Q99296

Q99383

Q99395

Alberti, et al. list

--------------------

P04147

P06634

P08004

P0C2I9

P0CX57

P0CX58

P11746

P12383

P14680

P18480

P18888

P18899

P19659

P20424

P20676

P22082

P22148

P22579

P23291

P23293

P24276

P24784

P25294

P25302

P25339

P25567

P25644

P25655

P27692

P28007

P29295

P31384

P32505

P32521

P32790

P32831

P32862

P32896

P32900

P33338

P33417

P34217

P34758

P34761

P35056

P35190

P35191

P35732

P37304

P38080

P38236

P38266

P38631

P38741

P38781

P38827

P38856

P38873

P38996

P39008

P39016

P39081

P39517

P39523

P39743

P39935

P39936

P40002

P40159

P40343

P40357

P40467

P40473

P40482

P40485

P40956

P40989

P41696

P43572

P43582

P45976

P45978

P47099

P47100

P47135

P48353

P48361

P48415

P48562

P48837

P49686

P49687

P50109

P50896

P53165

P53281

P53297

P53309

P53438

P53617

P53829

P53836

P53845

P53919

P80667

Q00539

Q00772

Q01477

Q01560

Q02199

Q02630

Q02773

Q02792

Q02796

Q03088

Q03390

Q03466

Q03735

Q03761

Q03825

Q03964

Q04214

Q04215

Q05672

Q05785

Q06251

Q06315

Q06337

Q06449

Q07684

Q08601

Q08831

Q08969

Q12034

Q12057

Q12085

Q12112

Q12118

Q12124

Q12141

Q12151

Q12266

Q12329

Q12391

Q12398

Q12489

Q12490

Q12518

Q12523

Q99383

'intersection' set of Harrison, et al and Alberti, et al. lists

---------------------------------------------------------------

P04147

P08004

P11746

P12383

P14680

P18480

P18888

P19659

P22082

P22148

P23291

P24276

P25302

P25339

P25644

P25655

P32790

P32831

P32862

P32896

P32900

P33338

P33417

P34217

P34758

P34761

P35732

P38080

P38741

P38781

P38873

P38996

P39008

P39081

P39523

P39935

P39936

P40002

P40357

P40467

P40473

P40485

P41696

P43572

P43582

P47135

P48361

P48562

P48837

P49686

P49687

P50109

P50896

P53165

P53281

P53438

P53829

P53919

Q00539

Q00772

Q02199

Q02630

Q02773

Q02792

Q03088

Q03390

Q03466

Q03735

Q03761

Q03825

Q05785

Q06251

Q06315

Q08601

Q08831

Q08969

Q12034

Q12057

Q12124

Q12151

Q12489

Q12490

Q12518

Q99383
