# Supplementary material for: Interaction Networks of Prion, Prionogenic and Prion-Like Proteins in Budding Yeast, and Their Role in Gene Regulation
Source: PLoS One. 2014 Jun 27;9(6):e100615. doi: 10.1371/journal.pone.0100615 (PMC4074094; doi:10.1371/journal.pone.0100615)
Supplement: Text S2 — Enrichments in Gene Ontology (GO) biological process categories for the EPD, EPN and NQP data sets. The format of each line is as follows (the n fields are numbered $1,$2,…,$n and tab delimited): $1 = data set (KP, EPD, EPN or NQP). $2 = GO category. $3 = description of the GO category in words. $4 = hypergeometric probability of the enrichment. For the NQP set, those GO biological process categories that are significantly enriched after a Holm-Bonferroni correction for multiple hypotheses, are listed. For the other data sets, none of the categories are significantly enriched after the Holm-Bonferroni correction. In these cases, any with P-value < = 1E−4 are listed. (DOC) [file pone.0100615.s004.doc]

Text S2: Enrichments in Gene Ontology (GO) biological process

categories for the EPD, EPN and NQP data sets

The format of each line is as follows (the n fields are numbered $1,$2,...,$n and

tab delimited):

$1 = data set (KP, EPD, EPN or NQP)

$2 = GO category

$3 = description of the GO category in words

$4 = hypergeometric probability of the enrichment

For the NQP set, those GO biological process categories that are significantly

enriched after a Holm-Bonferroni correction for multiple hypotheses, are listed.

For the other data sets, none of the categories are significantly enriched after

the Holm-Bonferroni correction. In these cases, any with P-value <= 1E-4 are listed.

EPD GO:0045893 positive regulation of transcription, DNA-templated 2.21E-5

EPD GO:0051173 positive regulation of nitrogen compound metabolic process 7.38E-5

EPD GO:2001057 reactive nitrogen species metabolic process 7.58E-5

EPD GO:0009628 response to abiotic stimulus 8.24E-5

EPD GO:0043620 regulation of DNA-templated transcription in response to stress 1E-4

EPN GO:0010468 regulation of gene expression 8.67E-6

EPN GO:0032774 RNA biosynthetic process 2.29E-5

EPN GO:0009893 positive regulation of metabolic process 1E-4

NQP GO:0006357 regulation of transcription from RNA polymerase II promoter 3.65E-35

NQP GO:0010468 regulation of gene expression 6.02E-33

NQP GO:0051254 positive regulation of RNA metabolic process 4.11E-32

NQP GO:0051252 regulation of RNA metabolic process 7.37E-31

NQP GO:0051173 positive regulation of nitrogen compound metabolic process 1.21E-30

NQP GO:0048518 positive regulation of biological process 1.29E-30

NQP GO:0009893 positive regulation of metabolic process 1.86E-30

NQP GO:0010628 positive regulation of gene expression 2.11E-30

NQP GO:0060255 regulation of macromolecule metabolic process 3.15E-30

NQP GO:0031325 positive regulation of cellular metabolic process 3.2E-30

NQP GO:0010604 positive regulation of macromolecule metabolic process 3.58E-30

NQP GO:2000112 regulation of cellular macromolecule biosynthetic process 4.52E-30

NQP GO:0006355 regulation of transcription, DNA-templated 9.51E-30

NQP GO:0045893 positive regulation of transcription, DNA-templated 1.07E-29

NQP GO:0050794 regulation of cellular process 2.34E-29

NQP GO:0050789 regulation of biological process 4.28E-29

NQP GO:0009889 regulation of biosynthetic process 4.44E-29

NQP GO:0031326 regulation of cellular biosynthetic process 4.44E-29

NQP GO:0019222 regulation of metabolic process 5.39E-28

NQP GO:0048522 positive regulation of cellular process 6.09E-28

NQP GO:0051171 regulation of nitrogen compound metabolic process 6.71E-28

NQP GO:0010557 positive regulation of macromolecule biosynthetic process 9.89E-28

NQP GO:0009891 positive regulation of biosynthetic process 1.22E-27

NQP GO:0031328 positive regulation of cellular biosynthetic process 1.22E-27

NQP GO:0006351 transcription, DNA-templated 1.35E-27

NQP GO:0031323 regulation of cellular metabolic process 1.84E-27

NQP GO:0080090 regulation of primary metabolic process 1.92E-27

NQP GO:0045944 positive regulation of transcription from RNA polymerase II promoter 3.38E-27

NQP GO:0065007 biological regulation 5.3E-24

NQP GO:0016070 RNA metabolic process 8.29E-17

NQP GO:0019438 aromatic compound biosynthetic process 9.45E-17

NQP GO:0018130 heterocycle biosynthetic process 4.88E-16

NQP GO:0044271 cellular nitrogen compound biosynthetic process 7.29E-16

NQP GO:1901362 organic cyclic compound biosynthetic process 2.28E-14

NQP GO:0043620 regulation of DNA-templated transcription in response to stress 2.39E-14

NQP GO:0043618 regulation of transcription from RNA polymerase II promoter in response to stress 2.39E-14

NQP GO:0090304 nucleic acid metabolic process 3.37E-13

NQP GO:0009059 macromolecule biosynthetic process 3.41E-13

NQP GO:0034645 cellular macromolecule biosynthetic process 1.45E-12

NQP GO:0036003 positive regulation of transcription from RNA polymerase II promoter in response to stress 2.83E-10

NQP GO:0061392 regulation of transcription from RNA polymerase II promoter in response to osmotic stress 4.15E-10

NQP GO:0044260 cellular macromolecule metabolic process 6.12E-10

NQP GO:0050896 response to stimulus 2.25E-9

NQP GO:0043170 macromolecule metabolic process 5.5E-9

NQP GO:0051716 cellular response to stimulus 9.22E-9

NQP GO:0034063 stress granule assembly 6.76E-8

NQP GO:0032197 transposition, RNA-mediated 9.34E-8

NQP GO:0000956 nuclear-transcribed mRNA catabolic process 9.9E-8

NQP GO:0009628 response to abiotic stimulus 1.11E-7

NQP GO:0006402 mRNA catabolic process 1.25E-7

NQP GO:0009991 response to extracellular stimulus 2.73E-7

NQP GO:0009605 response to external stimulus 2.73E-7

NQP GO:0032196 transposition 3E-7

NQP GO:0031670 cellular response to nutrient 3.22E-7

NQP GO:0051049 regulation of transport 3.82E-7

NQP GO:0071496 cellular response to external stimulus 4.4E-7

NQP GO:0031668 cellular response to extracellular stimulus 4.4E-7

NQP GO:0031667 response to nutrient levels 4.4E-7

NQP GO:0031124 mRNA 3'-end processing 4.43E-7

NQP GO:0042221 response to chemical 4.53E-7

NQP GO:0007584 response to nutrient 6.03E-7

NQP GO:0031669 cellular response to nutrient levels 7.08E-7

NQP GO:0006725 cellular aromatic compound metabolic process 7.38E-7

NQP GO:0071474 cellular hyperosmotic response 8.41E-7

NQP GO:0061393 positive regulation of transcription from RNA polymerase II promoter in response to osmotic stress 8.41E-7

NQP GO:0010558 negative regulation of macromolecule biosynthetic process 9.75E-7

NQP GO:0001558 regulation of cell growth 9.82E-7

NQP GO:0051050 positive regulation of transport 9.82E-7

NQP GO:0046890 regulation of lipid biosynthetic process 9.82E-7

NQP GO:0000436 carbon catabolite activation of transcription from RNA polymerase II promoter 1.2E-6

NQP GO:0046483 heterocycle metabolic process 1.42E-6

NQP GO:0006366 transcription from RNA polymerase II promoter 1.7E-6

NQP GO:0061416 regulation of transcription from RNA polymerase II promoter in response to salt stress 1.81E-6

NQP GO:0070887 cellular response to chemical stimulus 2.29E-6

NQP GO:2000113 negative regulation of cellular macromolecule biosynthetic process 2.3E-6

NQP GO:0000288 nuclear-transcribed mRNA catabolic process, deadenylation-dependent decay 2.3E-6

NQP GO:0072364 regulation of cellular ketone metabolic process by regulation of transcription from RNA polymerase II promoter 2.3E-6

NQP GO:0009890 negative regulation of biosynthetic process 2.72E-6

NQP GO:0031327 negative regulation of cellular biosynthetic process 2.72E-6

NQP GO:0006468 protein phosphorylation 3.24E-6
