# Supplementary material for: Interaction Networks of Prion, Prionogenic and Prion-Like Proteins in Budding Yeast, and Their Role in Gene Regulation
Source: PLoS One. 2014 Jun 27;9(6):e100615. doi: 10.1371/journal.pone.0100615 (PMC4074094; doi:10.1371/journal.pone.0100615)
Supplement: Text S3 — Gene Ontology biological process categories that have high membership, for the EPD and NQP data sets. The format of each line is as follows (the n fields are numbered $1,$2,…,$n, tab-delimited): $1 = Number of yeast proteins in the GO category. $2 = Number of proteins from the set (EPD or NQP) in the GO category. $3 = the fraction of the yeast proteins in the GO category that are in the set (EPD or NQP). $4 = GO biological process category. $5 = description of the GO category in words. $6 = hypergeometric probability of the enrichment. $7 = YES or NO for whether the enrichment passes the Holm-Bonferroni correction for multiple hypotheses. All enrichments with raw hypergeometric P-values < = 1E−4 are listed, if also most (> = 0.5) of the yeast proteins in thatGO biological process are included in the data sets. For the NQP set, those that are are significant after a Holm-Bonferroni correction are listed first. (DOC) [file pone.0100615.s005.doc]

Text S3: Gene Ontology biological process categories that

have high membership, for the EPD and NQP data sets

*The format of each line is as follows (the n fields are numbered $1,$2,…,$n, tab-delimited):

$1 = Number of yeast proteins in the GO category

$2 = Number of proteins from the set (EPD or NQP) in the GO category

$3 = the fraction of the yeast proteins in the GO category that are in the set (EPD or NQP)

$4 = GO biological process category

$5 = description of the GO category in words

$6 = hypergeometric probability of the enrichment

$7 = YES or NO for whether the enrichment passes the Holm-Bonferroni correction for multiple hypotheses.

All enrichments with raw hypergeometric P-values >=1E-3 are listed, if also most (>=0.5) of the yeast proteins in thatGO biological process are included in the data sets. For the NQP set, those that are are significant after a Holm-Bonferroni correction are listed first.

EPD set

=======

3 2 0.666667 GO:2001057 reactive nitrogen species metabolic process 7.58E-5 NO

NQP set

=======

5 5 1 GO:0061416 regulation of transcription from RNA polymerase II promoter in response to salt stress 1.81E-6 YES

10 9 0.9 GO:0061392 regulation of transcription from RNA polymerase II promoter in response to osmotic stress 4.15E-10 YES

8 7 0.875 GO:0034063 stress granule assembly 6.76E-8 YES

7 6 0.857143 GO:0071474 cellular hyperosmotic response 8.41E-7 YES

7 6 0.857143 GO:0061393 positive regulation of transcription from RNA polymerase II promoter in response to osmotic stress 8.41E-7 4.91E-5 YES

11 7 0.636364 GO:0072364 regulation of cellular ketone metabolic process by regulation of transcription from RNA polymerase II promoter 2.3E-6 YES

11 7 0.636364 GO:0000288 nuclear-transcribed mRNA catabolic process, deadenylation-dependent decay 2.3E-6 YES

18 9 0.5 GO:0000436 carbon catabolite activation of transcription from RNA polymerase II promoter 1.2E-6 YES

30 15 0.5 GO:0036003 positive regulation of transcription from RNA polymerase II promoter in response to stress 2.83E-10 YES

4 4 1 GO:0071456 cellular response to hypoxia 2.56E-5 NO

4 4 1 GO:0061404 positive regulation of transcription from RNA polymerase II promoter in response to increased salt 2.56E-5 NO

4 4 1 GO:0036251 positive regulation of transcription from RNA polymerase II promoter in response to salt stress 2.56E-5 NO

3 3 1 GO:0061422 positive regulation of transcription from RNA polymerase II promoter in response to alkalinity 3.61E-4 NO

3 3 1 GO:0060256 regulation of flocculation 3.61E-4 NO

3 3 1 GO:0051594 detection of glucose 3.61E-4 NO

3 3 1 GO:0035957 positive regulation of starch catabolic process by positive transcriptional regulation from RNA polII promoter 3.61E-4 NO 6 5 0.833333 GO:0071475 cellular hyperosmotic salinity response 1.02E-5 NO

5 4 0.8 GO:0061412 positive regulation of transcription from RNA polymerase II promoter in response to amino acid starvation 1.21E-4 3.68E-3 NO

5 4 0.8 GO:0045597 positive regulation of cell differentiation 1.21E-4 NO

5 4 0.8 GO:0031496 positive regulation of mating type switching 1.21E-4 NO

5 4 0.8 GO:0010455 positive regulation of cell fate commitment 1.21E-4 NO

5 4 0.8 GO:0001666 response to hypoxia 1.21E-4 NO

7 5 0.714286 GO:0070417 cellular response to cold 3.37E-5 NO

7 5 0.714286 GO:0031494 regulation of mating type switching 3.37E-5 NO

7 5 0.714286 GO:0010453 regulation of cell fate commitment 3.37E-5 NO

7 5 0.714286 GO:0009409 response to cold 3.37E-5 NO

6 4 0.666667 GO:0051094 positive regulation of developmental process 3.42E-4 NO

6 4 0.666667 GO:0045896 regulation of transcription during mitosis 3.42E-4 NO

6 4 0.666667 GO:0042304 regulation of fatty acid biosynthetic process 3.42E-4 NO

6 4 0.666667 GO:0001324 age-dependent response to oxidative stress involved in chronological cell aging 3.42E-4 NO

8 5 0.625 GO:0042538 hyperosmotic salinity response 8.46E-5 NO

8 5 0.625 GO:0005987 sucrose catabolic process 8.46E-5 NO

8 5 0.625 GO:0005985 sucrose metabolic process 8.46E-5 NO

7 4 0.571429 GO:0045764 positive regulation of cellular amino acid metabolic process 7.53E-4 NO

7 4 0.571429 GO:0034198 cellular response to amino acid starvation 7.53E-4 NO

7 4 0.571429 GO:0001306 age-dependent response to oxidative stress 7.53E-4 NO

7 4 0.571429 GO:0000289 nuclear-transcribed mRNA poly(A) tail shortening 7.53E-4 NO

7 4 0.571429 GO:0000117 regulation of transcription involved in G2/M transition of mitotic cell cycle 7.53E-4 NO (5462,7,390,4)

9 5 0.555556 GO:0072366 regulation of cellular ketone metabolic process by positive regulation of transcription from RNA polymerase II promoter 1.79E-4 NO

11 6 0.545455 GO:0009743 response to carbohydrate 4.33E-5 NO

12 6 0.5 GO:0061408 positive regulation of transcription from RNA polymerase II promoter in response to heat stress 8.14E-5 NO

12 6 0.5 GO:0006607 NLS-bearing protein import into nucleus 8.14E-5 NO

10 5 0.5 GO:0019217 regulation of fatty acid metabolic process 3.38E-4 NO

10 5 0.5 GO:0009749 response to glucose 3.38E-4 NO
