# Supplementary material for: Interaction Networks of Prion, Prionogenic and Prion-Like Proteins in Budding Yeast, and Their Role in Gene Regulation
Source: PLoS One. 2014 Jun 27;9(6):e100615. doi: 10.1371/journal.pone.0100615 (PMC4074094; doi:10.1371/journal.pone.0100615)
Supplement: Text S4 — Enrichments in Gene Ontology (GO) biological process categories for the protein interactions of the EPD, EPN and NQP data sets. The format of each line is as follows (the n fields are numbered $1,$2,…,$n and tab delimited): $1 = Number of yeast proteins in the GO category. $2 = Number of proteins in the GO category that are interacted with by the set. $3 = GO category. $4 = description of the GO category in words. $5 = hypergeometric probability of the enrichment. $6 = YES or NO for whether the enrichment passes the Holm Bonferroni correction for multiple hypotheses. For the NQP set, those GO biological process categories that are significantly enriched after a Holm-Bonferroni correction for multiple hypotheses, are listed. For the other data sets, none of the categories are significantly enriched after the Holm-Bonferroni correction. In these cases, any with P-value < = 1E−4 are listed. (DOC) [file pone.0100615.s006.doc]

Text S4: Enrichments in Gene Ontology (GO) biological process

Categories for the protein interactions of the EPD, EPN and NQP data sets

The format of each line is as follows (the n fields are numbered $1,$2,…,$n and tab delimited):

$1 = Number of yeast proteins in the GO category

$2 = Number of proteins in the GO category that are interacted with by the set

$3 = GO category

$4 = description of the GO category in words

$5 = hypergeometric probability of the enrichment

$6 = YES or NO for whether the enrichment passes the Holm-Bonferroni correction for multiple hypotheses.

For the NQP set, those GO biological process categories that are significantly enriched after

a Holm-Bonferroni correction for multiple hypotheses, are listed. For the other data sets, none

of the categories are significantly enriched after the Holm-Bonferroni correction. In these

cases, any with P-value <= 1E-4 are listed.

EPD set

=======

10 3 GO:0061158 3'-UTR-mediated mRNA destabilization 1.73247e-05 NO

EPN set

=======

13 3 GO:0051220 cytoplasmic sequestering of protein 2.36112e-05 NO

43 4 GO:0043488 regulation of mRNA stability 3.9563e-05 NO

22 3 GO:0061157 mRNA destabilization 1e-04 NO

NQP set

=======

83 26 GO:0034063 stress granule assembly 6.28772e-11 YES

43 18 GO:0043488 regulation of mRNA stability 2.43618e-10 YES

128 30 GO:0000184 nuclear-transcribed mRNA catabolic process, nonsense-mediated decay 5.03062e-09 YES

97 23 GO:0000290 deadenylation-dependent decapping of nuclear-transcribed mRNA 2.23198e-07 YES

99 23 GO:0017148 negative regulation of translation 3.27316e-07 YES

30 12 GO:0033673 negative regulation of kinase activity 4.25831e-07 YES

13 8 GO:0051220 cytoplasmic sequestering of protein 6.27764e-07 YES

13 8 GO:1900153 positive regulation of nuclear-transcribed mRNA catabolic process, deadenylation-dependent decay 6.27764e-07 YES

81 20 GO:0000956 nuclear-transcribed mRNA catabolic process 6.62999e-07 YES

37 13 GO:0045900 negative regulation of translational elongation 8.05389e-07 YES

22 10 GO:0061157 mRNA destabilization 9.65464e-07 YES

468 62 GO:0051028 mRNA transport 1.45246e-06 YES

126 25 GO:0006611 protein export from nucleus 2.22003e-06 YES

104 22 GO:0033962 cytoplasmic mRNA processing body assembly 2.93534e-06 YES
