# Supplementary material for: Interaction Networks of Prion, Prionogenic and Prion-Like Proteins in Budding Yeast, and Their Role in Gene Regulation
Source: PLoS One. 2014 Jun 27;9(6):e100615. doi: 10.1371/journal.pone.0100615 (PMC4074094; doi:10.1371/journal.pone.0100615)
Supplement: Text S5 — Enriched Gene Ontology (GO) biological process categories for the NQP interactors of the three hubs LSM4, PUB1 and NUP100. The format of each line is as follows (the n fields are numbered $1,$2,…,$n and tab delimited): $1 = Number of yeast proteins in the GO category. $2 = Number of proteins in the GO category that are interactors of the specific KP/EPD protein examined. $3 = GO category. $4 = description of the GO category in words. $5 = hypergeometric probability of the enrichment. The enrichments that are significant after multiple-hypothesis correction with the Holm-Bonferroni method are listed. (DOC) [file pone.0100615.s007.doc]

Text S5: Enriched Gene Ontology (GO) biological process categories for the NQP

interactors of the three hubs LSM4, PUB1 and NUP100

The format of each line is as follows (the n fields are numbered $1,$2,...,$n and

tab delimited):

$1 = Number of yeast proteins in the GO category

$2 = Number of proteins in the GO category that are interactors of the specific KP/EPD

protein examined

$3 = GO category

$4 = description of the GO category in words

$5 = hypergeometric probability of the enrichment

The enrichments that are significant after multiple-hypothesis correction with the

Holm-Bonferroni method are listed.

LSM4:

=====

14 6 GO:0017148 negative regulation of translation 3.13E-12

14 7 GO:0000956 nuclear-transcribed mRNA catabolic process 3.53E-10

14 8 GO:0010608 posttranscriptional regulation of gene expression 3.62E-10

14 6 GO:0032269 negative regulation of cellular protein metabolic process 1.58E-9

14 4 GO:0034063 stress granule assembly 1.89E-9

14 9 GO:0044265 cellular macromolecule catabolic process 8.82E-9

14 4 GO:0000290 deadenylation-dependent decapping of nuclear-transcribed mRNA 8.86E-9

14 3 GO:0045947 negative regulation of translational initiation 1.34E-7

14 11 GO:0010468 regulation of gene expression 1.98E-7

14 5 GO:0070925 organelle assembly 2.98E-7

14 7 GO:0019439 aromatic compound catabolic process 6.11E-7

14 3 GO:0033962 cytoplasmic mRNA processing body assembly 7.48E-7

14 7 GO:0016071 mRNA metabolic process 1.94E-6

PUB1:

=====

13 8 GO:0010608 posttranscriptional regulation of gene expression 1.58E-10

13 4 GO:0017148 negative regulation of translation 9.2E-8

13 3 GO:0043488 regulation of mRNA stability 2.3E-6

NUP100:

=======

10 7 GO:0051028 mRNA transport 2.79E-11

10 6 GO:0006406 mRNA export from nucleus 2.78E-10

10 7 GO:0051169 nuclear transport 1.6E-9

10 5 GO:0006606 protein import into nucleus 8.32E-9

10 9 GO:0051649 establishment of localization in cell 6.41E-8

10 4 GO:0016973 poly(A)+ mRNA export from nucleus 7.07E-8

10 8 GO:0016482 cytoplasmic transport 1.46E-7

10 10 GO:0006810 transport 1.75E-7

10 4 GO:0006997 nucleus organization 2.27E-7

10 3 GO:0006607 NLS-bearing protein import into nucleus 9.67E-7

10 3 GO:0006409 tRNA export from nucleus 1.26E-6
